# Downregulated TICAM1 is a prognostic biomarker and associated with immune tolerance of Wilms tumor patients

**DOI:** 10.1186/s12920-022-01326-5

**Published:** 2022-08-06

**Authors:** Zhiyi Lu, Fengyin Sun

**Affiliations:** Department of Pediatric Surgery, Qilu Hospital of Shandong University, Jinan 250000, Shandong, China

**Keywords:** Wilms tumor, Immune tolerance, TICAM1

## Abstract

**Background:**

TIR domain containing adaptor molecule 1 (TICAM1) is a coding gene participating in immune and inflammation responses to malignant cells. However, the role of TICAM1 in Wilms tumor (WT) is rarely known.

**Materials and methods:**

The expression level of TICAM1 was calculated in the WT TARGET cohort and validated using the GSE66405 cohort. The Kaplan–Meier method was employed to investigate the potential clinical value of TICAM1 and the association between its expression level and clinical features. The influence of TICAM1 on immune infiltration was examined by ESTIMATE, CIBERSORT and MCPcounter algorithms. IC50 of chemotherapeutic drugs was calculated by “pRRophetic” R package.

**Results:**

TICAM1 was downregulated in WT patients with worse prognosis and a more advanced clinical stage. Moreover, a low expression level of TICAM1 contributed to less immune cell infiltration, few protective immune cells and more antitumor immune cells.

**Conclusions:**

TICAM1 exerts a significant impact on the prognosis, progression and immune infiltration condition of WT.

**Supplementary Information:**

The online version contains supplementary material available at 10.1186/s12920-022-01326-5.

## Introduction

Wilms tumor (WT) is a kidney cancer and common malignant tumor of the urinary system in children. Radical resection and targeted chemotherapy can increase the 5-year survival rate of WT patients from 75 to 90%, effectively improving the patient prognosis and raising their expectation level of a good prognosis [1, 2]. Normal kidneys undergo normal mesenchymal-epithelial transitions during development, while a difficult transition from mesenchymal to epithelial states is often observed in WT patients [3]. The recurrence and metastasis mechanisms of WT are so intricate that it cannot be eliminated completely. There is abundant genetic research into the pathogenesis of WT, but the pathogenesis, metastasis and recrudescence of WT have not been fully elucidated at the genetic level. [4] Unlike other cancers, WT is prone to young patients, and it is insufficient to just improve their five-year survival rate. Thus, it is critical to identify a novel biomarker for the diagnosis and prognosis predication of WT.

Toll–IL-1 receptor (TIR) domain-containing adaptor molecule-1 (TICAM1), also known as TRIF, is an adaptor protein which participates in TLR3 mediated proinflammatory cytokine and interferon (IFN) responses and induces IFN generation [5]. Type I interferon plays an essential role in the body’s specific immune responses to malignant cells, like the tumor cells [6, 7]. TLR3-TICAM1 pathway can mediate the formation of mature myeloid dendritic cells and render tumor-protecting macrophages to acquire tumoricidal properties [8]. Dendritic cells can promote adaptive immune responses, such as anticancer immunity, while tumor infiltration of polarized M2 macrophages damages adaptive immunity and promotes tumor progression [9]. According to previous studies, activating TLR3-TICAM1 pathway could inhibit the progression of tumor cells in multiple myeloma [10]. Tumor microenvironment affects cancer progression. In tumor microenvironment, there are not just a variety of immune cells, but inflammatory cells also account for a large part. Some immune cells also participate in inflammatory responses [11]. However, the mechanism of TICAM1 underlying the development of WT has not been studied in depth.

In this study, RNA-sequencing (RNA-seq) data extracted from TARGET and GEO databases were used to analyze the association between TICAM1 and prognosis of WT. The results demonstrated that the expression of TICAM1 was downregulated in WT tissue compared with that in adjacent normal tissue. TICAM1 was correlated with overall survival of WT patients. Meanwhile, TICAM1 expression was closely related to clinicopathological factors. Further analysis suggested that TICAM1 facilitated immune infiltration, which meant that TICAM1 had an immunosuppression effect on WT tissue. Taken above, TICAM1 is a valuable novel prognostic biomarker of WT.

## Materials and methods

### Data source

The WT TARGET cohort (https://ocg.cancer.gov/) was downloaded from Xena Browser (http://xena.ucsc.edu/). The expression data (FPKM) and corresponding clinical data of 132 WT patients were downloaded, and FPKM data were transformed into transcripts per million (TPM) for further analysis by R software. The expression data included 126 tumor samples and 6 normal samples. Clinical data of WT patients consist of transcriptome data, survival datas and clinical features. Patients who survived longer than thirty days were selected. Moreover, the GSE66405 dataset was downloaded from GEO database (https://www.ncbi.nlm.nih.gov/geo/;). All data used were acquired from TCGA and GEO. So neither the ethical approval nor the informed consent of the patients was required.

### Identifying and validating the expression of TICAM1 in WT tissue

The expression level of TICAM1 was analyzed by comparing tumor and normal tissue in the TARGET cohort, and it was validated in the GSE66405 cohort. Afterwards, the expression level of TICAM1 in other cancers was examined using the pan-cancer data from the TCGA database.

### Survival analysis

126 WT patients were divided into the high expression group and the low expression group by the best cutoff values, which were calculated by “survminer” R package. To demonstrate the prognostic value of TICAM1, the Kaplan–Meier (KM) curve was applied to verifying the independence of the signature and was visualized by using the “survival” R package and “survminer” R package. A P value of < 0.05 represented significant difference.

### Relationship between TICAM1 and clinical data of WT in the TARGET cohort

To explore the clinical value of TICAM1, the association between the expression level of TICAM1 and clinicopathological factors (i.e., the tumor stage, tumor relapse, gender, and age) was checked. The univariate cox analysis was conducted to identify independent significant prognosis factors.

### Functional enrichment analysis by GSEA

In order to clarify deeper mechanisms underlying the role of TICAM1 in the progression of WT, GSEA software (4.2.3) was used to analyze hallmark gene sets. 126 patients were classed into the high expression group and the low expression group according to the expression levels of TICAM1, and the high expression group contained 51 samples and the low expression group contained 75 samples. The groupings here were consistent with the groupings for the survival analysis. *P* < 0.05 and NES > 2.00 were indicators of a close relationship between TICAM1 expression levels and WT progression.

### Immune infiltration analysis

To evaluate the immune infiltration level in WT tissue, the Stromal, Immune and Estimate scores of WT and normal tissue were calculated by the ESTIMATE algorithm and compared. CIBERSORT and MCPcounter algorithms were used to analyze the immune cell distribution in two groups and the correlation of TICAM1 expression levels with immune cells.

### Chemotherapeutic drug sensitivity analysis

Drug sensitivity comparison between the two groups revealed that downregulated TICAM1 expression aggravated drug resistance. The half-maximal inhibitory concentration (IC50) was an indicator of the response rate of chemotherapeutic drugs to tumor cells. IC50 was calculated by “pRRophetic” R package.

### Statistical analysis

Unpaired and paired t tests were performed to check the differences of TICAM1 expression using R software. Cox analysis in SPSS was used to determine the independent prognostic risk factors of WT patients. Survival differences were analyzed by the log-rank test and the results were mapped into a KM curve. Correlation analysis was carried out using the Pearson correlation approach. R packages used included “survival”, “limma”, “survminer”, “ggplot2”, “pRRophetic”, “cibersort”, “mcpcounter” and “estimate”. A P value of < 0.05 was considered statistically significant.

## Results

### Identification of lower TICAM1 expression in WT

**Fig. 1:**
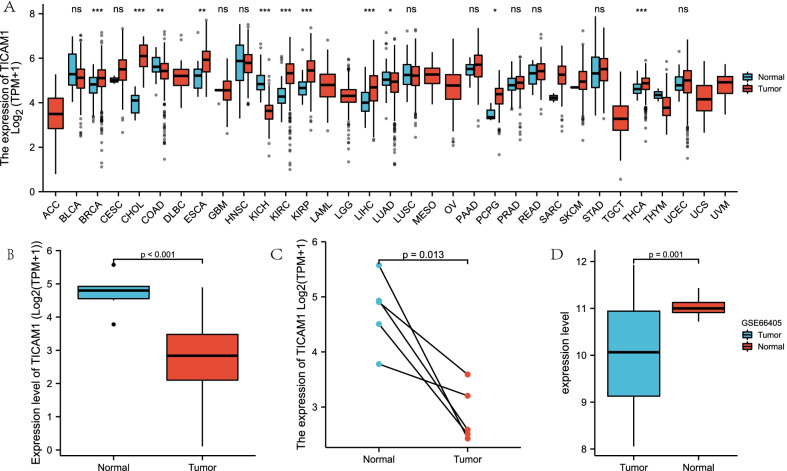
Expression level of TICAM1. TICAM1 expression level analysis in pan-cancer (**A**). TICAM1 expression in WT and normal tissue in TARGET cohort (**B**). TICAM1 paired expression analysis in WT and normal tissue in TARGET cohort (**C**). TICAM1 expression in external validation set GSE66405 (**D**)

The expression level of TICAM1 in diverse cancers was analyzed using the pan-cancer data, and the results indicated that TICAM1 was downregulated in some cancers like KICH and LUAD, and upregulated in such cancers as KIRC, STAD, etc. (Fig. 1A) The clinical characteristics of the TARGET cohort are shown in Table 1. In this cohort, the expression of TICAM1 mRNA in WT tissue was significantly lower than that in normal tissue, according to the paired and unpaired t test results (Fig. 1B, C). This finding was verified by the external validation set GSE66405 (Fig. 1D).

**Table 1:** Clinical features of the WT TARGET cohort

	TARGET(N = 126)
Gender	
Female	70(55.56%)
Male	56(44.44%)
Age	
< 3 years	33(26.19%)
> 3 years	93(73.80%)
Stage	
I-II	68(53.97%)
III-IV	58(46.03%)
Evenet	
Relapse/progression	101(80.06%)
None	25(19.84)
TICAM1	
High-expression`	51(40.48%)
Low-expression	75(59.52%)

### TICAM1 is a prognostic biomarker

**Fig. 2:**
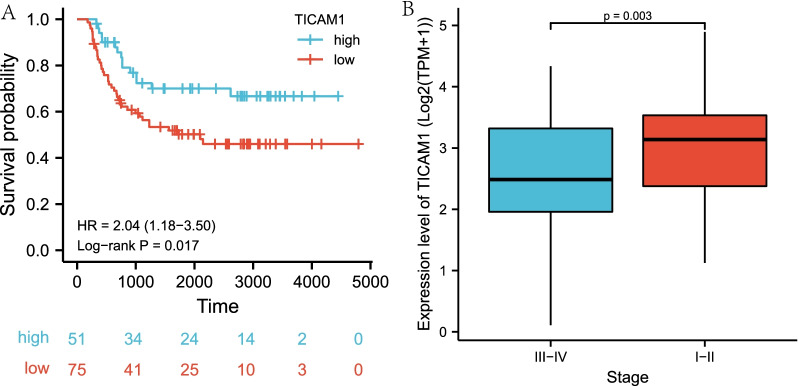
Survival analysis and clinical feature analysis. K-M plot (**A**). The association of TICAM1 and stage (**B**)

The best cutoff was determined by R software as 3.137. A total of 51 samples with a best cutoff value exceeding 3.137 were included in the high expression group, and the remaining 75 samples belonged to the low expression group. In order to explore the prognostic value of TICAM1, a KM plot was plotted for the WT TARGET cohort. The results showed that the low expression group had a lower survival probability than the high expression group (Fig. 2A). Besides, the association of TICAM1 expression levels and clinical characteristics between two groups was studied. It was found that TICAM1 expression levels were not statistically significantly correlated with all clinicopathological factors, except the tumor stage (Fig. 2B). In WT patients staged III-IV, the expression level of TICAM1 was lower than that in WT patients staged I-II, which suggested that a higher stage accompanied by low expression of TICAM1. Finally, the univariate cox analysis was carried out to analyze the association of patient survival and clinical data. The results indicated that the expression of TICAM1, tumor stage, adverse event rate and gender were independent prognosis factors of WT (Table 2).

**Table 2:** Univariate COX regression analysis of clinical features

id	univariate Cox			
	HR	HR.95L	HR.95H	*P *value
Gender	1.92	1.115	3.308	0.019
Age	1.131	0.614	2.083	0.694
Event	31.54	2.174	457.578	*P* < 0.01
Stage	2.553	1.463	4.457	*P* < 0.01
TICAM1	0.49	0.27	0.892	0.02

### Functional enrichment analysis

**Fig. 3:**
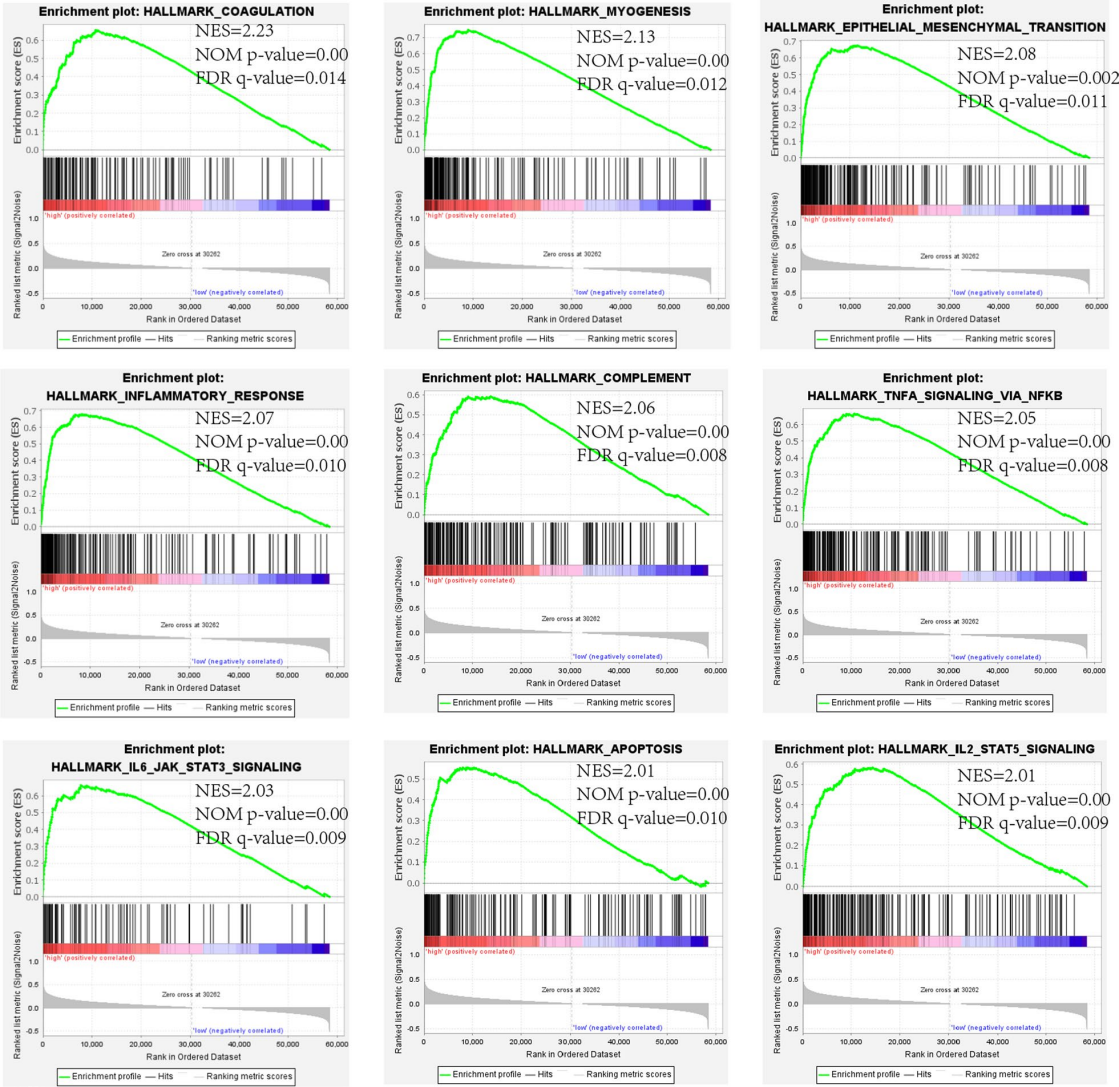
Top 9 significant results in Hallmark sets

To further confirm the role of TICAM1 in WT progression, metastasis and relapse, hallmark gene sets were analyzed using the GSEA software. GSEA results demonstrated that in the group with high TICAM1 expression, the coagulation, myogenesis, epithelial-mesenchymal transition (EMT), inflammatory response, complement, NFKB-mediated TNFA signaling, IL6-JAK-STAT3 signaling, and apoptosis pathways were significant (Fig. 3). They were immunity and inflammation related pathways. However, the low expression group had significantly highly expressed E2F targets and MYC targets, both of which participated in progression of most cancers (Additional file 1).

### Immune infiltration analysis

**Fig. 4:**
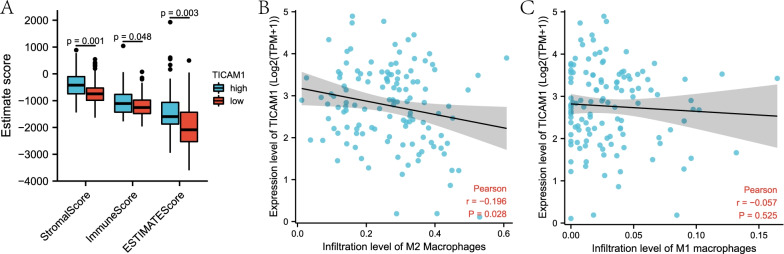
The result of ESTIMATE and CIBERSORT. ESTIMATE (**A**), M2 macrophages in CIBERSORT (**B**), M1 macrophages (**C**)

**Fig. 5:**
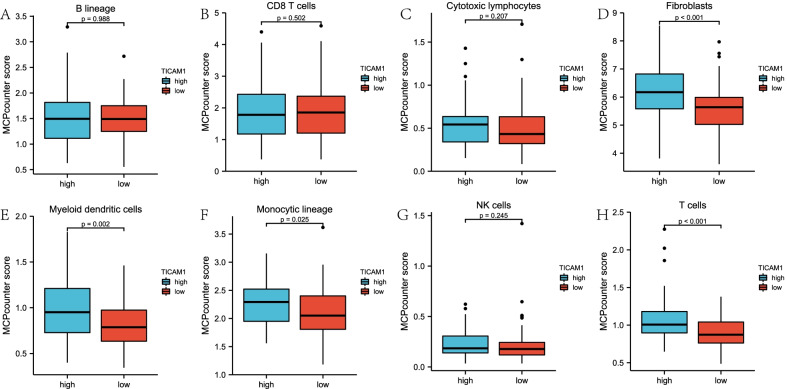
The results of MCPcounter. B lineage (**A**), CD8 T cells (**B**), Cytotoxic lymphocytes (**C**), Fibroblasts (**D**), Myeloid dentritic cells (**E**), Monocytic lineage (**F**), NK cells (**G**), T cells (**H**)

To explore whether the TICAM1 expression level was associated with immunity, ESTIMATE, CIBERSORT and MCPcounter algorithms were used to evaluate WT immune infiltration. The ESTIAMTE algorithm result indicated that the low expression group had significantly lower Stromal, Immune and Estimate scores than the high expression group (Fig. 4A). The CIBERSORT algorithm result suggested that the expression of TICAM1 mRNA was negatively associated with M2 macrophages, but not correlated with M1 macrophages (Fig. 4B, C). The MCPcounter algorithm result proved that T cells, monocytic lineage cells, myeloid dendritic cells and fibroblasts in the low expression group were significantly fewer than those in the high expression group (Fig. 5A–H).

### IC50 score

**Fig. 6:**
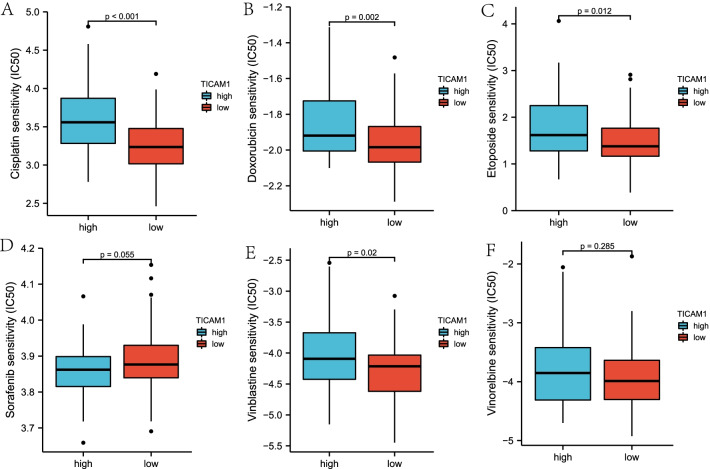
Distribution of IC50 scores. Cisplatin (**A**), Doxorubicin (**B**), Etoposide (**C**), Sorafenib (**D**), Vinblastine (**E**), Vinorelbine (**F**)

IC50 is a biomarker of the response rate of chemotherapeutic drugs to tumor cells. The IC50 scores of chemotherapy drugs in the low and high expression groups of the WT TARGET cohort were predicted. The result indicated that compared with the high expression group, the low expression group had lower IC50 scores of Cisplatin, Doxorubicin, Etopside and Vinblastine, but a higher IC50 score of Sorafenib (Fig. 6A–F). Thus, TICAM played a vital role in the sensitivity of tumor cells to chemotherapeutic drugs.

## Discussion

Genetic prognostic markers are essential to the diagnosis and prognosis of cancers, and identifying more prognostic markers can improve our understanding of WT. Although the diagnosis and prognosis of WT have been dramatically improved by standardized surgical treatments such as early diagnosis and preoperative chemotherapy, chemotherapeutic drug resistance and immune tolerance still pose a major challenge. [12] Therefore, further exploration of the mechanism of WT is warranted. The pan-cancer analysis results show that TICAM1 is either highly or lowly expressed in other cancers, but in the WT TARGET cohort and external validation cohort GSE66405, it is confirmed that TICAM1 expression in WT tissue is significantly lower than that in normal tissue (*p* < 0.05). Although TICAM1 has been extensively studied in inflammation responses and some cancers such as prostate cancer [13], breast cancer [14], and colorectal cancer [15], there is still a lack of knowledge about the role of TICAM1 in WT progression. The effect of immune microenvironment of WT has not been clarified yet, but our study suggests that TICAM1 affects immune cell distribution in WT tissue. In the present study, the influence of TICAM1 on WT is explored for the first time.

TICAM1, also known as a TIR domain-containing adapter inducing IFN-β (TRIF) protein, is a coding protein and an innate immune system protein [16]. Increasing evidence demonstrates that TICAM1 participates in TLR3-TICAM1, TLR4-TICAM1 signaling pathways and type I IFN induction. TLR3-TICAM1 and TLR4-TICAM1 pathways and type I IFNs are important inflammation response pathways that mediate specific immune responses to malignant cells [17]. Moreover, TLR3-TICAM1 pathway promotes the development of important immune cells to resist tumor, such as dendritic cells and macrophages. TICAM1 has been found to participate in immune responses and promote apoptosis in other cancers [18]. The role of TICAM1 in tumors is tissue-specific and its expression varies in different tumors. TICAM1 may up-regulate and down-regulate the expression of different genes via the same pathway in different tumors, thus forming diversified gene expression profiles and playing divergent roles in tumor development and evolution. Previous studies proved that activating TLR3/TICAM1 signaling could suppress multiple myeloma progression [10]. Based on the above findings, the role TICAM1 in WT progression, metastasis and relapse requires further research.

In this study, the group with low TICAM1 expression has worse overall survival and a more advanced clinical stage. It indicates that TICAM1 acts as a suppressor of WT and has a predictive value. This finding is consistent with that of the previous study, which concluded that activating TLR3/TICAM1 signaling could arrest the progression of multiple myeloma. The GSEA functional enrichment analysis results reveal that inflammation responses, apoptosis, NFKB-mediated TNFA signaling and coagulation are significantly more intense in the group with high expression of TICAM1. It implies that a low expression level of TICAM1 in WT can promote WT progression. The enriched apoptosis in the high expression group and inhibited apoptosis in the low expression group suggests that curbing apoptosis encourages WT progression. TLR3-TICAM1 signaling is an inflammation response pathway, and in WT, downregulated TICAM1 may prevent inflammation responses to tumor cells. TICAM1 can also enhance anticancer immunity by stimulating NFKB generation, and low TICAM1 expression in WT may weaken anticancer immunity and accelerate the progression of WT cells [19, 20]. TICAM1 not only participates in inflammation responses but also affects the immune system. Immune cells and cytokines facilitate the occurrence, progression, metastasis, and relapse of cancers, so an immune infiltration algorithm is used to evaluate the association of TICAM1 with immune infiltration and the distribution of immune cells. The ESTIMATE algorithm analysis results show that lower expression of TICAM1 leads to lower immunity. Two groups with different TICAM1 expression levels have different immune conditions, and the low expression group presents higher immune resistance. According to the CIBERSORT algorithm analysis results, the expression of TICAM1 mRNA is negatively related to M2 macrophages. That is to say, a lower expression level of TICAM1 results in more M2 macrophages. Researchers have established that elevated M2 macrophages suggests enhanced immunosuppressive functions, so it will further lead to lower expression of TICAM1 and poorer prognosis in WT patients [21]. As indicated by the MCPcounter algorithm results, T cells, monocytic lineage cells, myeloid dendritic cells and fibroblasts are decreased in the low expression group. The decline of these cells will intensify immunosuppressive activities. T cells are vital to antitumor immunity, and TICAM1 boosts the production and maturation of dendritic cells, which are important immune cells [5]. TLR3, which is positively correlated to TICAM1, is also downregulated in WT tissue. TLR3-TICAM1 participates in induction of myeloid dendritic cells and antitumor macrophages [8]. However, low expression of the TLR3-TICAM1 pathway reduces the dendritic cells and increases M2 macrophages, thereby weakening immune responses to tumor cells and stimulating tumor cells to progress. Immunosuppression is associated with rapid tumor progression, chemotherapeutical drug resistance and tumor metastasis [22]. The number of type I IFNs produced is decreased in WT patients with a low expression level of TICAM1. Nevertheless, type I IFNs play an essential role in specific immune responses to malignant cells, such as tumor cells. Therefore, TICAM1 has great significance in initiating immune and inflammatory responses.

The importance of TICAM1 is comprehensively illustrated in this study, but it also has some limitations. First, this study is based on bioinformatics analyses, and the data are originated from public databases, so it lacks the verification of experiment data. Second, the immunity is assessed by algorithms, and the immunity of WT patients in clinical practice should be examined by experimental observation. Further in-depth studies are required to address the relationship between the expression level of TICAM1 and immune infiltration.

In conclusion, TICAM1 is downregulated in WT, which is significantly associated with a poor survival prognosis and a more advance clinical stage. Downregulated TICAM1 may be involved in tumor associated immune responses and tumor progression. Besides, according to basic pathway mechanisms and bioinformatics prediction results, downregulated TICAM1 is likely to promote the generation of M2 macrophages and weaken immune responses to tumor cells. Therefore, TICAM1 is a potential therapeutic target in WT.

## Supplementary Information

**Additional file 1.** The result of GSEA.

## Data Availability

The raw data of this study are derived from the TARGET database (https://ocg.cancer.gov/) and GEO database (https://www.ncbi.nlm.nih.gov/geo/), the specifc sample is TARGET-WT, GSE66405, which are provided on public databases.

## References

[1] Long CJ, Mittal S, Kolon TF (2022). Expanding the use of nephron-sparing surgery for wilms tumor. J Natl Compr Canc Netw.

[2] Pater L, Melchior P, Rube C, Cooper BT, McAleer MF, Kalapurakal JA, Paulino AC (2021). Wilms tumor. Pediatr Blood Cancer.

[3] Tamimi Y, Ekuere U, Laughton N, Grundy P (2008). WNT5A is regulated by PAX2 and may be involved in blastemal predominant Wilms tumorigenesis. Neoplasia.

[4] Cui WW, Sun YL, Chen C, Feng RR, Xu W, Meng JJ, Zhang K (2020). LncRNA CRNDE promotes the development of Wilms' tumor by regulating microRNA-424. Eur Rev Med Pharmacol Sci.

[5] Seya T, Oshiumi H, Sasai M, Akazawa T, Matsumoto M (2005). TICAM-1 and TICAM-2: toll-like receptor adapters that participate in induction of type 1 interferons. Int J Biochem Cell Biol.

[6] Tao S, Zhu L, Lee P, Lee WM, Knox K, Chen J, Di YP, Chen Y (2012). Negative control of TLR3 signaling by TICAM1 down-regulation. Am J Respir Cell Mol Biol.

[7] Le Naour J, Galluzzi L, Zitvogel L, Kroemer G, Vacchelli E (2020). Trial watch: TLR3 agonists in cancer therapy. Oncoimmunology.

[8] Matsumoto M, Funami K, Oshiumi H, Seya T (2013). Toll-IL-1-receptor-containing adaptor molecule-1: a signaling adaptor linking innate immunity to adaptive immunity. Prog Mol Biol Transl Sci.

[9] Mantovani A, Sozzani S, Locati M, Allavena P, Sica A (2002). Macrophage polarization: tumor-associated macrophages as a paradigm for polarized M2 mononuclear phagocytes. Trends Immunol.

[10] Chen Y, Zhao J, Li D, Hao J, He P, Wang H, Zhang M (2018). TRIM56 suppresses multiple myeloma progression by activating TLR3/TRIF signaling. Yonsei Med J.

[11] Khandia R, Munjal A (2020). Interplay between inflammation and cancer. Adv Protein Chem Struct Biol.

[12] Huang J, Zhang Y, Zhen Z, Lu S, Zhu J, Wang J, Sun F, Liu Z, Gao Y, Li H (2020). The prognosis of prechemotherapy blastemal predominant histology subtype in Wilms tumor: a retrospective study in China. Pediatr Blood Cancer.

[13] Deveci Ozkan A, Kaleli S, Onen HI, Sarihan M, Guney Eskiler G, Kalayci Yigin A, Akdogan M (2020). Anti-inflammatory effects of nobiletin on TLR4/TRIF/IRF3 and TLR9/IRF7 signaling pathways in prostate cancer cells. Immunopharmacol Immunotoxicol.

[14] Guney Eskiler G, Deveci Ozkan A, Kaleli S, Bilir C (2019). Inhibition of TLR4/TRIF/IRF3 Signaling Pathway by Curcumin in Breast Cancer Cells. J Pharm Pharm Sci.

[15] Firmal P, Shah VK, Pant R, Chattopadhyay S (2022). RING finger protein TOPORS modulates the expression of tumor suppressor SMAR1 in colorectal cancer via the TLR4-TRIF pathway. Mol Oncol.

[16] Hyland EM, Webb AE, Kennedy KF, Gerek Ince ZN, Loscher CE, O'Connell MJ. Adaptive evolution in TRIF leads to discordance between human and mouse innate immune signaling. *Genome Biol Evol* 2021, **13**(12). 10.1093/gbe/evab268 PMC8691055 34893845

[17] Ullah MO, Sweet MJ, Mansell A, Kellie S, Kobe B (2016). TRIF-dependent TLR signaling, its functions in host defense and inflammation, and its potential as a therapeutic target. J Leukoc Biol.

[18] Liu R, Liu X, Song M, Qi Y, Li H, Yang G, Shan S (2021). Cyprinus carpio TRIF Participates in the Innate Immune Response by Inducing NF-kappaB and IFN Activation and Promoting Apoptosis. Front Immunol.

[19] Takaki H, Oshiumi H, Sasai M, Kawanishi T, Matsumoto M, Seya T (2009). Oligomerized TICAM-1 (TRIF) in the cytoplasm recruits nuclear BS69 to enhance NF-kappaB activation and type I IFN induction. Eur J Immunol.

[20] Taniguchi K, Karin M (2018). NF-kappaB, inflammation, immunity and cancer: coming of age. Nat Rev Immunol.

[21] Wang N, Liu W, Zheng Y, Wang S, Yang B, Li M, Song J, Zhang F, Zhang X, Wang Q (2018). CXCL1 derived from tumor-associated macrophages promotes breast cancer metastasis via activating NF-kappaB/SOX4 signaling. Cell Death Dis.

[22] Gajewski TF, Schreiber H, Fu YX (2013). Innate and adaptive immune cells in the tumor microenvironment. Nat Immunol.

