# Supplementary material for: Downregulated TICAM1 is a prognostic biomarker and associated with immune tolerance of Wilms tumor patients
Source: BMC Med Genomics. 2022 Aug 6;15:174. doi: 10.1186/s12920-022-01326-5 (PMC9356447; doi:10.1186/s12920-022-01326-5)
Supplement: Supplementary file 1 — Additional file 1. The result of GSEA. [file 12920_2022_1326_MOESM1_ESM.pdf]

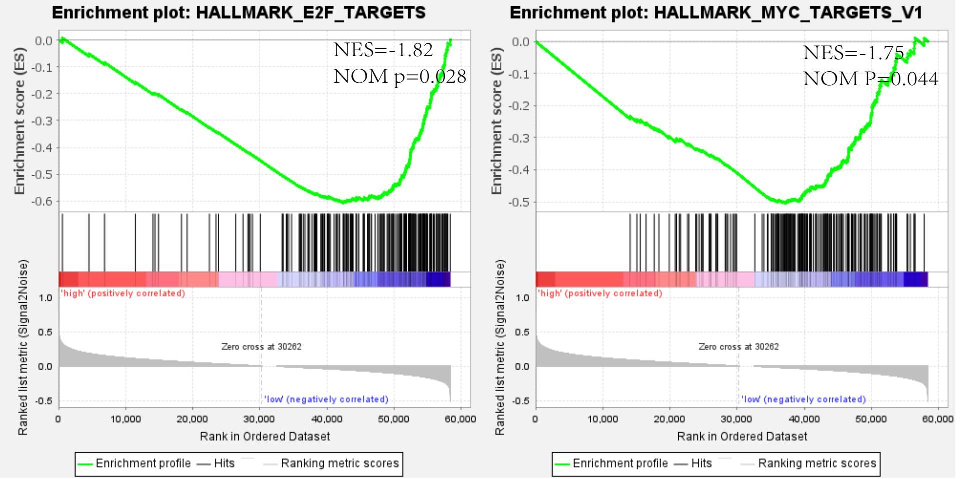

Supplemental file 1: The result of GSEA.
